# Internal Status of Visibly Opaque Black Rubbers Investigated by Terahertz Polarization Spectroscopy: Fundamentals and Applications

**DOI:** 10.3390/polym11010009

**Published:** 2018-12-21

**Authors:** Makoto Okano, Shinichi Watanabe

**Affiliations:** Department of Physics, Faculty of Science and Technology, Keio University, 3-14-1, Hiyoshi, Kohoku-ku, Yokohama, Kanagawa 223-8522, Japan; watanabe@phys.keio.ac.jp

**Keywords:** black rubber composites, terahertz polarization spectroscopy, carbon black fillers, internal strain, percolation conductivity

## Abstract

We discuss the internal status of rubber composites consisting of an insulating rubber matrix and conductive carbon black (CB) fillers (“black rubber”) using polarization-sensitive terahertz time-domain spectroscopy (THz-TDS). The black rubber composites under stretched conditions exhibit a large optical anisotropy or birefringence in the terahertz regime. From systematic studies, it is revealed that the large birefringence of black rubbers is due to the orientation distribution of anisotropically shaped CB aggregates in the rubber matrix and the orientation distribution is strongly linked to the mechanical deformation of the black rubber. A model simulation based on this relation between deformation and reorientation allows conversion of the birefringence (*optical*) information into strain (*mechanical*) information. In addition, the spectroscopic information obtained using the THz-TDS technique is useful to evaluate the changes in the internal conductive filler network caused by the mechanical deformation. Our findings demonstrate that the terahertz polarization spectroscopy is a promising nondestructive inspection method for contactless investigation of the internal condition of black rubber composites.

## 1. Introduction

Rubbers are popular and indispensable polymeric materials in modern society because of their specific characteristics, for instance a low elastic modulus, or a low glass transition temperature [1]. Actually, rubber products are already so common that we can hardly spend a single day without them. In particular, rubber composites that consist of a rubber matrix and functional additives (the so-called “fillers”) are very useful materials for various industrial applications [2]. By adding different functional fillers, the rubber composites gain specific properties such as high durability, conductivity, or corrosion resistance [2]. The rubber composite consisting of a rubber matrix and conductive carbon black (CB) fillers (hereafter referred to as “black rubber”) is one of the most useful class of rubber composites and is widely utilized for various rubber products such as tires, dampers, sealing materials and so forth. In addition, the application of rubber composites is expected to increase further. In that regard, it is rather surprising that the probably most fundamental question remains unresolved, that is, how do functional fillers relate to the properties of the rubber composite? For a better understanding of the role of the functional fillers in the composites, it is essential to investigate the internal condition of the rubber–filler composites. Consequently, numerous studies have been devoted to the characterization of the influence of the functional fillers on the properties of the rubber composites [3,4,5,6,7,8,9,10]. Recent technological advances pathed the way for revealing the internal mechanics of the rubber–filler composites by several techniques such as small angle X-ray scattering (SAXS) including theoretical simulations [5,6], transmittance electron microscopy (TEM) [7] and their combinations [8,9,10]. On the other hand, it is well known that the optical technique is the one of the most useful and effective techniques to investigate the properties of polymeric materials [11,12,13,14]. Unfortunately, since most rubber composites like black rubbers are visibly opaque, the conventional method based on visible light cannot be utilized for investigating the internal state of the rubber–filler composites.

Because terahertz light allows us to evade this problem and has clear advantages over the abovementioned techniques, the terahertz technology is expected to provide powerful tools for investigating polymeric materials. Actually, since terahertz light possesses a relatively low photon energy (below one hundred meV), it can penetrate the visibly opaque polymeric materials and directly probe the characteristic vibration mode inherent to polymers [15,16,17,18,19,20,21,22,23,24,25,26]. In recent years, owing to significant improvements in terahertz technology, terahertz radiation has been widely utilized as an advanced type of laser technology [27,28]. In particular, the terahertz time-domain spectroscopy (THz-TDS) based on ultrafast pulsed lasers has been widely used, because of its characteristic feature which allows us to directly obtain spectroscopic information for the complex refractive index, that is, the complex dielectric functions and conductivity. Thus, many terahertz applications involving polymers have been developed; the fingerprint spectroscopy [29], the differentiation between the enantiomer and stereo-complex [30], the determination of the glass transition temperature [31] and the monitoring of the phase transition [26,32,33]. Very recently, the polarization-sensitive (PS) THz-TDS has attracted interest from the viewpoints of both fundamental research [34,35,36,37,38,39,40] and potential terahertz applications [41,42,43,44]. For instance, the PS THz-TDS enables the inspection of the birefringent properties [15,25,26,38,42] and the filler orientation in glass-fiber reinforced polymers [38]. As such, terahertz radiation has many advantages.

Although various applications of terahertz technology in fundamental and applied polymer science have been proposed during the past two decades, it seems that its potential is still not fully utilized. In addition, because of the difficult operation of THz-TDS systems and complex analysis of the THz-TDS data, not all researchers in the field of polymer science may be aware of the manifold capabilities of THz-TDS. Thus, it should be quite instructive to demonstrate the effectiveness and characteristics of the terahertz technology from the viewpoint of an expert of terahertz spectroscopy but strong focus on the applications important to polymer scientists. In this invited review, to highlight the usefulness of the terahertz technology for polymer sciences, as one of the best examples, we summarize our recent researches regarding the internal condition of black rubber composites with the PS THz-TDS techniques [25,42,43]. We firstly revealed the fundamental physics of the anisotropic optical responses of the stretched black rubbers in the terahertz frequency range [25]. Based on the revealed fundamental knowledge about the terahertz optical responses of black rubbers, two applications of the PS THz-TDS to nondestructively characterize the internal status of black rubbers have been demonstrated; the internal triaxial strain inspection [42] and the sensing of the internal conductive filler network [43]. Our researches based on terahertz technology focus on the emerging candidates for investing the internal condition of black rubbers.

## 2. Materials and Methods

Here, we briefly explain the samples and the experimental setup that are referred to during the discussions in this review. We used two types of black rubbers with conductive CB fillers: a fluoroelastomer (FKM) (V-100, Togawa Rubber) and a styrene butadiene rubber (SBR). In particular, for the experiment in Section 3.3, we prepared three SBR samples with different amounts of CB fillers (0, 15 and 30 wt %.) In addition, as a sample without any CB fillers, we prepared a natural rubber (NR) sample (AGS-14, Wakisangyo Co., Ltd). The thicknesses of all samples were approximately 1 mm. For the optical measurements, we cut the rubber sheets into rectangular specimens with a long axis of 50 mm and a short axis of 20 mm.

**Figure 1:**
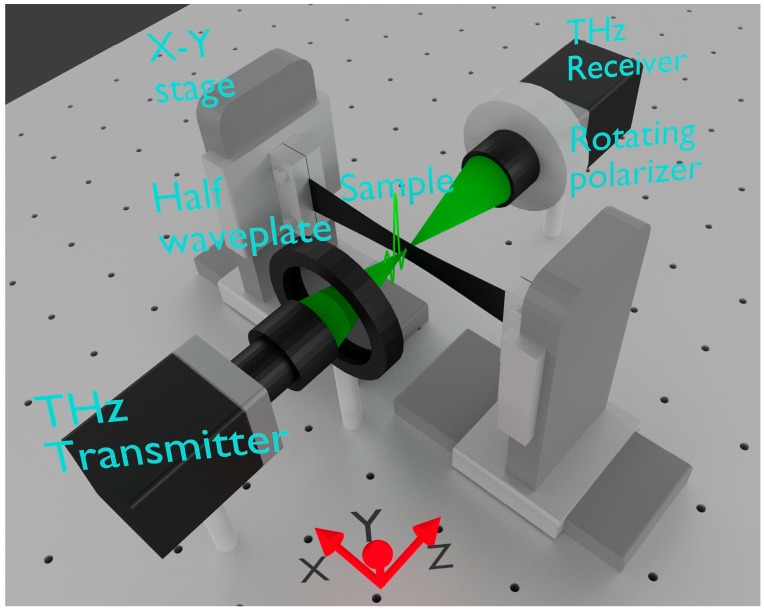
A three-dimensional illustration of the handmade polarization-sensitive terahertz time-domain spectroscopy (PS THz-TDS) system for investigating the optical responses of black rubber composites. This setup enables strain imaging of stretched rubber samples.

We performed the PS THz-TDS experiments with a handmade PS THz-TDS system consisting of a commercially available high-speed THz-TDS system (T-ray 5000, Terametrix, Luna Inc.) and a handmade polarimetry setup, which is based on the rotating polarizer technique [25,37,45,46]. Figure 1 shows a three-dimensional illustration of our PS THz-TDS system. The commercial terahertz pulse transmitter and receiver (which are based on photoconductive antennas with a frequency range from 0.1 to 2.0 THz and a frequency resolution of 12.5 GHz) were used with a measurement repetition rate of ~ 1 kHz. We mounted a wire-grid polarizer (EWG40-I, Origin Co., Ltd.) on a hollow-shaft motor (HM2853E18HA, Technohands Co., Ltd.) placed in front of the terahertz receiver and rotated it with a frequency of 40 Hz. This enables one complete polarization measurement within 25 milliseconds, which is relatively fast compared to the conventional terahertz polarization spectroscopy [34,35,36,37,38,39,40]. More technical details of our PS THz-TDS system and the analytical method can be found in Ref. [25]. To perform measurements of the rubber samples under stretched conditions and also imaging measurements, we clamped the two short edges of the sample with aluminum blocks to the left and the right. These aluminum blocks are mounted on the separately electrically controlled X–Y stages (KXL06100-N1-C and KXL06075-N1-C (Suruga Seiki Co., Ltd.) for the X and Y stages, respectively), which allow us to control the stretching condition and the measurement position on the sample. In the present experiments, we applied uniaxial strain along the *x*-axis, where we define the *x*-, *y*- and *z*-axes as the directions parallel to the long and short edges of the sample and the direction along the terahertz propagation, respectively. We carefully measured the spot size on the sample by the knife-edge method and estimated a size of 2–3 mm for the range 0.2–0.4 THz. All optical measurements were carried out at room temperature in normal atmosphere.

In the experiments discussed below, we obtain the birefringent properties, that is, the degree of the birefringence Δ*n* and the angle of the slow optic axis *θ* with respect to the *x*-axis by analyzing the change in the polarization state of the terahertz light on a Poincaré sphere [25]. By using the Poincaré sphere representation, we can easily obtain the birefringent properties of samples by comparing the Stokes vectors (which represent the polarization information of the light) with and without samples. There are several important works regarding the description of polarized light using the Poincaré sphere representation [47,48,49]. More details of our analytic method based on this representation are provided in Ref. [25].

## 3. Experimental Results

**Figure 2:**
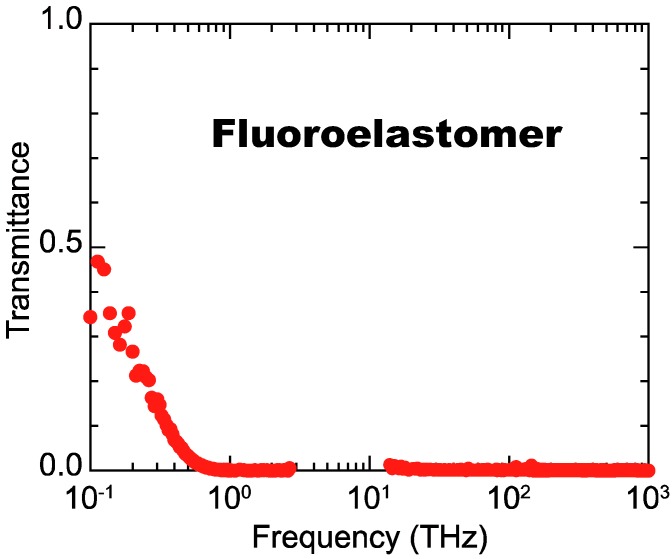
Transmittance spectrum of a 1-mm thick FKM composite ranging from the terahertz to the visible light regime. Reproduced from M. Okano et al. Sci. Rep. **6**, 39079 (2016) [25]. Licensed under CC BY 4.0.

First, we clarify why the terahertz spectroscopy is suited for investigating the internal condition of black rubber composites. A brief explanation is given with the transmittance property of the FKM sample as an example. Figure 2 shows the transmittance spectrum of the FKM sample with a thickness of 1 mm for frequencies ranging from the terahertz regime to visible light. The nonzero transmission for light with frequencies below 1 THz (corresponding to “terahertz light”) can penetrate the FKM rubber whereas the black rubber is hardly transparent for light with frequencies above 1 THz (including mid-, near-infrared and visible light). It is well known that the strong absorption in the region above 1 THz is mainly due to the absorption of the conductive CB fillers [19], which behave similar to the conductive carbon nanotube fillers [17,18,20,21,50]. This means that only terahertz light can be effectively utilized to probe the internal condition of black rubbers while the conventional methods based on mid-, near-infrared and visible light are not suited for this purpose. Therefore, we have intensively studied the fundamental physics that determine the optical responses of black rubbers at terahertz frequencies and explored several unique applications based on our findings. Below, we explain the fundamentals and applications for terahertz polarization spectroscopy.

### 3.1. Large Birefringence of Black Rubber Composites in the Terahertz Frequency Range

When utilizing the PS THz-TDS in investigations of the internal condition of black rubber composites, it is important to know what kind of information is obtained by the polarization-dependent optical responses in the terahertz frequency range. Thus, we initially investigated the polarization dependence of the optical responses of the black rubbers under various stretching conditions and tried to interpret the responses with an appropriate model [25]. We found that only black rubber composites with conductive CB fillers show a strain-induced terahertz birefringence that is one order of magnitude larger than that observed in the visible range. From comprehensive studies and a comparison between experimental results and theoretical calculations, we revealed that the anisotropic CB filler orientation is the reason for the large birefringence in the black rubber composites. For practical purposes, we determined the relationship between the birefringence and the internal filler orientation, which is one of the most important information for rubber composites.

**Figure 3:**
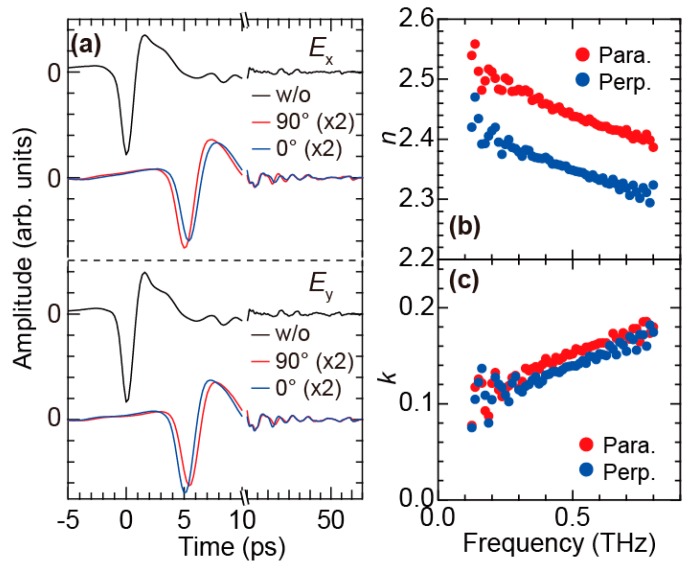
(**a**) *x*- (top panel) and *y*-components (bottom panel) of the terahertz E-field time-domain waveform without (black curves) and with the FKM sample for sample angles of 0° (blue curves) and 90° (red curves); (**b**) The real and (**c**) imaginary part of the complex refractive index parallel and perpendicular to the slow optic axis derived from the time-domain waveform in (a). Reproduced from M. Okano et al. Sci. Rep. **6**, 39079 (2016) [25]. Licensed under CC BY 4.0.

Figure 3a shows the polarization dependence of the terahertz electric-field (E-field) time-domain waveform obtained without and with the FKM sample under the unstretched condition. In this section, we only show the experimental data obtained at the center of the stretched samples. The time origin *t* = 0 ps is defined by the main peak of the E-field waveform without the sample for each polarization. We performed the PS THz-TDS measurements for the FKM sample with two different angles between the long sample edge and the *x*-axis, 0° and 90°, as shown with the blue and red curves in Figure 3a, respectively. The *x*- and *y*-components of the E-field time-domain waveform without the sample are almost the same. The E-field time-domain waveforms obtained with the FKM sample are delayed compared to those without the sample, because the refractive index of the sample is larger than that for air (unity). Moreover, the *x*(*y*)-component of the E-field waveform obtained for the sample angle 0°(90°) is delayed compared to the orthogonal component. This clearly indicates that an anisotropy in the refractive index exists in the FKM sample even under unstretched condition.

To quantitatively evaluate the anisotropy in the refractive index, we calculated the complex refractive index spectra of the FKM sample parallel and perpendicular to the slow optic axis. The slow optic axis is determined from the time-domain waveform shown in Figure 3a by using the Poincaré sphere representation [25,47,48,49]. In the THz-TDS, the complex refractive index spectrum ($\tilde{N}\left(\omega \right)=n\left(\omega \right)+ik\left(\omega \right)$) is calculated by the complex transmission spectrum that is defined as ${\tilde{E}}_{\mathrm{sample}}\left(\omega \right)/{\tilde{E}}_{\mathrm{ref}}\left(\omega \right),$ where ${\tilde{E}}_{\mathrm{sample}\left(\mathrm{ref}\right)}\left(\omega \right)$ is the frequency-domain Fourier component of the terahertz time-domain waveform obtained with(without) sample. The detailed calculation method is described in Ref. [28]. Figure 3b,c show the complex refractive index spectra of the FKM sample parallel and perpendicular to the slow optic axis, respectively. For both directions, we find that the real part of the complex refractive index, corresponding to the refractive index *n*, exhibits a monotonic decrease as the frequency increases. This frequency-dependent behavior is usually observed in the rubber–conductive-carbon-filler composites [17,18,19,20,21,50]. In addition, the difference between the refractive indices parallel and perpendicular to the slow optic axis is insensitive to the frequency and maintains an almost constant value (~0.1) within the measured terahertz frequency range. We note that the observed difference in the refractive indices parallel and perpendicular to the slow optic axis, that is, the birefringence in the terahertz regime is quite large (~0.1) compared to that observed for visible light (<0.01) [1]. Meanwhile, the imaginary part of the complex refractive index, which is proportional to the absorption coefficient, monotonically increases with the optical frequency and also shows a slight anisotropy. 

**Figure 4:**
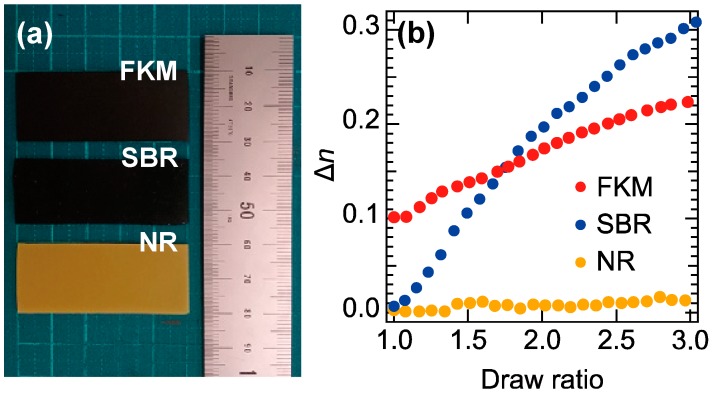
(**a**) Photograph of the FKM, SBR and NR samples; (**b**) Draw ratio dependence of the birefringence of the FKM (red circles), SBR (blue circles) and NR (orange circles) samples. Part of this figure is reproduced from M. Okano et al. Sci. Rep. **6**, 39079 (2016) [25]. Licensed under CC BY 4.0.

In order to understand the origin of the large birefringence in the FKM, we measured two additional types of the rubber samples: one is the SBR with CB fillers and the other is the NR without any CB fillers. Figure 4a shows a photograph of the three samples used for the measurements. The colors of the 1-mm thick FKM and SBR samples are black due to the addition of the CB fillers, whereas the color of the 1-mm thick NR sample is close to yellow. Figure 4b shows the draw ratio (DR) dependence of the birefringence of the three rubber samples. Here, we define DR as the ratio of the sample length after stretching to the initial length. From the refractive index spectra recorded for all samples (not shown here), we found that each sample’s birefringence is almost insensitive with respect to the frequency below 0.5 THz. Thus, we averaged their birefringence values over the range 0.2–0.3 THz and plotted them in Figure 4b. In the FKM sample, the birefringence is clearly different from zero at DR = 1 (unstretched condition) and monotonically increases above 0.2 as the DR increases from 1 to 3. On the other hand, in the SBR and NR samples, the birefringence is almost zero at DR = 1. As the DR increases, both the birefringence of the SBR sample and the NR sample monotonically increase but their slopes are quite different. At DR = 3, the birefringence of the SBR sample reaches a value larger than 0.3, while that of the NR sample only becomes approximately 0.01. From this measurement, we found that only black rubber materials with CB fillers exhibit a large birefringence! This implies that the large birefringence observed at terahertz frequencies is a result of the CB fillers addition and distribution.

In the following, we explain how these CB fillers can induce such a large birefringence. Although each CB particle is an almost isotropic sphere, it is well known that the CB fillers form anisotropically shaped aggregates inside the rubber matrix [7,51]. The anisotropic shape causes an anisotropy in the conductivity of the CB aggregate and thus these aggregates possess anisotropic dielectric functions. In addition, experiments have clearly shown that conductive carbon materials such as metallic carbon nanotubes with anisotropic dielectric functions induce optical anisotropy [50]. Therefore, we attribute the origin of the birefringence to the anisotropically shaped CB aggregates. This assignment naturally explains the reason for a relatively small birefringence obtained with visible light as reported previously [1]. Because rubber materials with conductive carbon fillers cannot transmit visible light, the birefringence of the rubber materials without conductive carbon fillers have been measured with visible light [1]. In this situation, the measured birefringence of rubbers mainly reflects the alignment of the rubber molecules. Because the anisotropy in the conductivity of the insulating rubber molecule is smaller than that of the conductive CB fillers, the relatively small birefringence of rubbers have been obtained with visible light compared with a large birefringence observed at terahertz frequencies. 

Our finding that the anisotropically shaped CB aggregate is the origin of the birefringence also provides an intuitive explanation of the DR dependence of the birefringence shown in Figure 4b: the orientation distribution of the CB aggregates governs the degree of the birefringence. For instance, in case of a randomly oriented condition, the birefringence is almost zero because the anisotropic optical response of each CB aggregate is cancelled by other aggregates with different orientation. Meanwhile, a perfect alignment of all CB aggregates causes a constructive summation of their optical anisotropies, resulting in a large birefringence. It has been reported that the stretching of a deformable sample induces a modulation of the orientation of the inclusions and aligns them more or less along the stretching direction [52]. Thus, the zero birefringence of the SBR sample at DR = 1.0 corresponds to a random orientation of the CB fillers and the strain-induced orientation of the CB fillers results in an increase of the birefringence with the DR as shown in Figure 4b. In addition, the large birefringence of the unstretched FKM sample indicates that the CB fillers are already partially oriented even under the unstretched condition. This initial preferential orientation is probably a result of the fabrication process. Because the rubber sheet is thinned by using rollers during the fabrication process, the CB aggregates may align normal to the roller axis. These results imply that the birefringence might be utilized an indicator for the degree of orientation of the conductive fillers with anisotropic conductivity.

**Figure 5:**
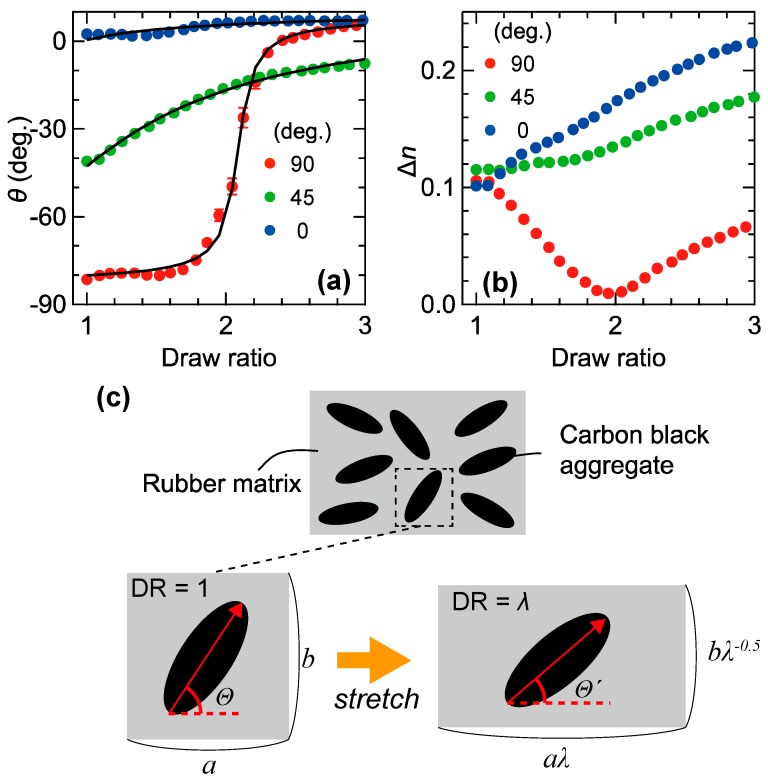
Draw ratio dependence of (**a**) the angle of the slow optic axis and (**b**) the degree of the birefringence of three FKM samples with initial orientation angles of 0° (blue circles), 45° (green circles) and 90° (red circles); (**c**) Schematic of our model for the change in the orientation of the CB aggregates due to mechanical stretching. Here, λ is defined as the draw ratio. Part of this figure is reproduced from M. Okano et al. Sci. Rep. **6**, 39079 (2016) [25]. Licensed under CC BY 4.0.

To clarify the validity of the abovementioned model and further understand the optical anisotropic responses of black rubber composites at terahertz frequencies, we investigated the DR dependence of three FKM samples with different initial angles between the slow optic axis and the stretching direction. Since the CB fillers in the FKM sample are already partially oriented along a certain direction even in the unstretched condition, we can prepare FKM samples with different angles (here, 0°, 45° and 90°) between the initial preferential angle of the slow optic axis and the long axis of the sample by cutting the FKM sheet along various directions. Figure 5a,b show the DR dependences of the angle of the slow optic axis and the degree of the birefringence, respectively. In the FKM sample with an initial angle of 0°, the birefringence monotonically increases as the DR increases and the angle of slow optic axis maintains approximately 0°. On the other hand, the sample with an initial angle of 90° exhibits a clearly different behavior. Here, the degree of birefringence first approaches zero as DR increases from 1 to ~2.0 and then starts to increase again. The corresponding angle of the slow optic axis first maintains approximately 90° for a DR below ~ 2 then, suddenly rotates to 0° around DR = 2 and finally reaches a stable value of approximately 0° above DR = 2. In the FKM sample with an initial angle of 45°, the degree of the birefringence shows a behavior that is similar to that observed from the sample with an initial angle of 0°, while the angle of slow optic axis slowly approaches 0° as the DR increases.

From comparison with the theoretical results of a Monte-Carlo (MC) simulation, we found that these behaviors can be interpreted by a simple mixture model. The schematic illustration of the simple mixture model before and after stretching is shown in Figure 5c. In this model, for simplicity, we assume that all CB aggregates can be represented by the same rigid uniaxial ellipsoid and an affine uniaxial deformation with a Poisson’s ratio of 0.5. We clarified that the latter condition is almost satisfied at the center of the stretched FKM sample by the separate measurements [25]. It is considered that the angle of the major axis of each CB aggregate, which corresponds to the slow optic axis, changes from a certain value Θ to Θ’ due to the stretching process of studied sample. The general relation between the Θ to Θ’ of an arbitrary aggregate can be expressed by [52]

(1) $$\Theta^{\prime }=\tan^{-1}\left(\lambda^{-\frac{3}{2}}\tan\Theta \right)$$

where λ is the draw ratio. Because the average direction of the major axes of the CB aggregates corresponds to the angle of the slow optic axis of the black rubber composite, we can determine the DR dependence of the black rubber’s angle of the slow optic axis by calculating and averaging the directions of the aggregates with a MC simulation. The detailed calculation procedure has been described in the Supplementary Information of Ref. [25]. The calculation results for one million samples are shown with the black curves in Figure 5a. All three experimental curves are well reproduced by the calculations with same initial orientation distribution but different initial preferential orientation angle. This good agreement between the experiment and the calculation strongly suggests that our simple mixture model well describes the essential mechanism of the optical responses of the black rubbers.

### 3.2. Inspection of the Internal Triaxial Strain of Black Rubber Composites

In the previous section, we found that the birefringent property is directly related to the mechanical deformation of the black rubbers via the strain-induced orientation distribution of the CB aggregates, which can be well described by MC simulations. A natural extension of these experiments is the imaging of the internal triaxial strain of stretched black rubbers. We demonstrated fast two-dimensional mapping of the internal strain of black rubbers by employing terahertz polarization spectroscopy [42]. Since the inspection of the internal condition of rubber composites is essential for preventing fatal accidents caused by large internal strain or fatigue [53,54], our new nondestructive and contactless inspection method is valuable for many purposes.

First, we briefly explain the procedure required for converting the birefringent information to strain information. In the PS THz-TDS experiments, we can obtain three types of information from the polarization-dependent time-domain terahertz E-field waveform with and without sample; namely the degree of birefringence, the angle of the slow optic axis [25,42] and the sample thickness at each measurement point [42,55]. Here, we assume that the *z*-axis is one of the principle strain axes because the sample thickness is thin. Consequently, the shear strain along the *z*-axis direction is considered to be zero. Thus, the strain tensor Λ, which needs to be evaluated, can be written as present in Equation (2):

(2) $$\Lambda =\;\left(\begin{array}{ccc} \epsilon_{xx} & \epsilon_{xy} & 0\\ \epsilon_{xy} & \epsilon_{yy} & 0\\ 0 & 0 & \epsilon_{zz} \end{array}\right),$$

where the first and second subscript of each strain component represent the normal direction of the surface where strain acts and the direction of the strain. Since the sample thickness is evaluated from spectral analysis in the THz-TDS measurement [42,55], $\epsilon_{zz}$ is directly derived from the ratio of the thickness before deformation to that after deformation. On the other hand, $\epsilon_{xx}$, $\epsilon_{yy}$ and $\epsilon_{xy}$ are derived from the comparison between the experimentally obtained birefringent properties (Δ*n*, *θ*) and the theoretical calculation results based on the MC simulation. In the case when the birefringence of the sample is almost zero under the unstretched condition, Δ*n* and *θ* are proportional to the degree and the direction of strain, respectively. In this situation, we firstly determine the diagonalized principal strain tensor  Λ ′ by comparison between experimentally obtained Δ*n* and calculated Δ*n* based on the MC simulation and then we convert Λ′ into Λ using *θ* with the following relationship.

(3) $$\Lambda =R\left(-\theta \right)\;\Lambda \;\prime R\left(\theta \right)=\left(\begin{array}{ccc} \cos\theta & -\sin\theta & 0\\ \sin\theta & \cos\theta & 0\\ 0 & 0 & 1 \end{array}\right)\left(\begin{array}{ccc} \epsilon_{1} & 0 & 0\\ 0 & \epsilon_{2} & 0\\ 0 & 0 & \epsilon_{3} \end{array}\right)\left(\begin{array}{ccc} \cos\theta & \sin\theta & 0\\ -\sin\theta & \cos\theta & 0\\ 0 & 0 & 1 \end{array}\right).$$

Utilizing this conversion procedure, we can evaluate the triaxial strain tensor shown in Equation (2). More details of the conversion procedure have been described in Ref. [42].

Please note the difference between the MC simulations performed in Section 3.1 and Section 3.2. In Section 3.1, we only measured the center of the stretched rubber sample. In this situation, we can assume a Poisson’s ratio of 0.5. However, in the experiment described in this section, we measured various positions on the stretched rubber. In this situation, we have to take into account for deformations that do not correspond to uniaxial deformation with a Poisson’s ratio of 0.5, because the deformation constraints depend on the spatial position. Thus, we calculated the DR dependence of the birefringent properties for various deformation conditions and utilized them to convert the birefringent properties to internal strain. 

**Figure 6:**
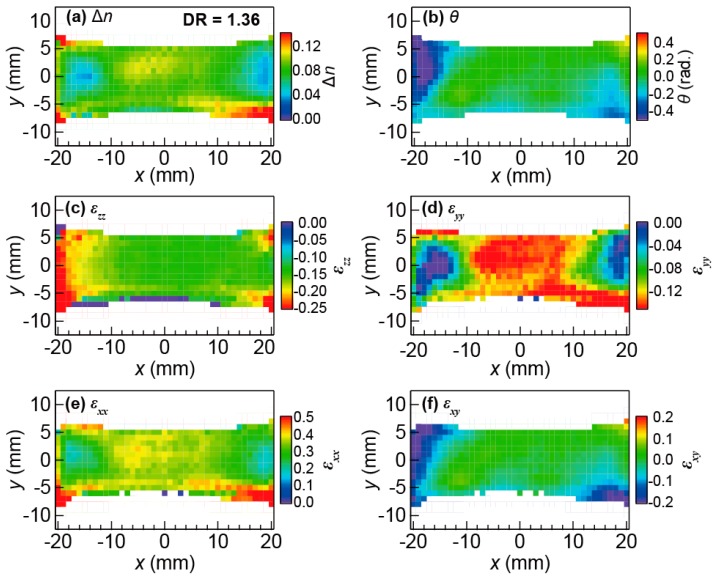
The spatial mapping of (**a**) the degree of the birefringence and (**b**) the angle of the slow optic axis of the SBR sample with a CB amount of 15 wt % for DR = 1.36; (**c**–**f**) The internal triaxial strain images of $\epsilon_{xx}$, $\epsilon_{yy}$, $\epsilon_{xy}$ and $\epsilon_{zz}$, respectively, derived from (a) and (b). Reproduced from A. Moriwaki et al. APL Photon. **2**, 106101 (2017) [42]. Licensed under CC BY 4.0.

Figure 6a,b show the two-dimensional mapping of the birefringent properties of a SBR sample with a CB amount of 15 wt % at DR = 1.36. In this experiment, the sample is stretched along the *x*-axis by applying external uniaxial stress. Δ*n* clearly shows a spatial inhomogeneity, that is, the birefringence near the center of the sample is large (~0.1) while it is small near the edges close to the clamps (near *x*~ ± 20 mm). Meanwhile, *θ* is almost constant zero except in the area near the clamps. Note that the map of Δ*n* for this sample at DR = 1 (not shown here) is almost everywhere zero and *θ* shows a strong fluctuation [42], indicating that the CB aggregates are randomly oriented in case of no external stress. On the other hand, the results shown in Figure 6a,b, that is, the large value of Δ*n* and the convergence of *θ* to zero clearly reveal a re-orientation of the CB aggregates towards the stretching direction.

Figure 6c–f show the internal triaxial strain images of the SBR sample for DR = 1.36, obtained from the birefringent images shown in Figure 6a,b with the help of MC simulations. All strain images show a similar tendency in the spatial distribution; a clear difference between the values in the center and the left and right edges is observed. This tendency reflects different constraining conditions. Hereafter, we explain the experimental results in terms of the different constraints. Firstly, $\left|\epsilon_{\mathrm{zz}}\right|$ is larger than $\left|\epsilon_{\mathrm{yy}}\right|$ at both edges, because the mechanical clamp prevents the shrinkage of the sample along the *y*-direction. Secondly, although $\epsilon_{\mathrm{xx}}$ possesses a spatial distribution due to the changing constraints, the average value of $\epsilon_{\mathrm{xx}}$ is almost 0.36 as expected from the DR. This fact verifies the validity of our inspection method. Thirdly, $\epsilon_{\mathrm{xy}}$ exhibits a clear asymmetric distribution, because we cannot fix the sample in a perfectly symmetrical way with the aluminum clamps.

For further verification of our method and concretization of its characteristics, we compared the spatial distribution of the strain with that obtained by conventional inspection of the surface strain with visible light, which is the so-called digital image correlation (DIC) method [56,57,58]. The comparison (not shown here) proves the validity of the internal triaxial strain inspection based on PS THz-TDS [42]. In addition, we verified important differences between the strain images obtained by PS THz-TDS measurements and the DIC method, especially in the edge area. We concluded that these differences reflect the fact that the PS THz-TDS-based method evaluates the internal strain whereas the DIC method evaluates the surface strain. Finally, we emphasize that the PS THz-TDS-based method has a clear advantage over the conventional DIC method, because the former method enables visualization of the triaxial strain (in contrast to the two-dimensional strain image for DIC) and does not require any pre-processing, which is essential for the DIC method. Thus, our developed method is a promising candidate for nondestructive inspection of internal strain in a contactless manner.

### 3.3. Evaluation of the Internal Conductive Filler Network based on the Effective Medium Theory

So far, we focused on the internal strain condition of the black rubber composites. Of course, useful internal information of the black rubbers is not restricted to strain information. For example, the condition of the internal CB filler network has also important technical implications. Since the internal filler network governs the optical, electrical and mechanical properties of rubber–filler composites [59,60], the information regarding the internal filler network would enable us to design and fabricate better rubber products. Usually, electrical measurements are utilized to investigate the internal conductive network [61,62,63,64,65,66,67]. However, since the electrical measurement requires fabrication of electrodes on the sample, the change in the internal network that is induced by mechanical stretching is difficult to evaluate. Meanwhile, since terahertz spectroscopy enables a contactless evaluation of the conductivity, we can investigate the strain-induced changes in the internal filler network without need of any additional sample processing. In addition, our PS THz-TDS technique enables investigation of the anisotropic conductive nature under stretched conditions, which is useful to unveil the mechanism of the electrical conduction in the black rubber composites.

**Figure 7:**
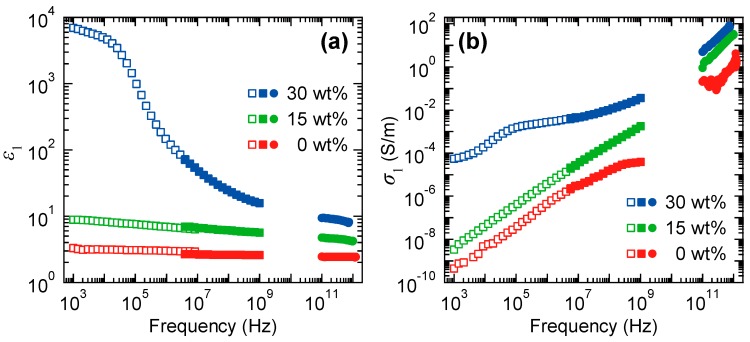
Frequency dependence of the real part of (**a**) the dielectric function and (**b**) the conductivity for the SBR samples with different CB amounts of 0 (red symbols), 15 (green symbols) and 30 wt % (blue symbols). The filled and open squares correspond to the data measured by two impedance analyzers with different measuring ranges and the filled circles correspond to those obtained by the THz-TDS measurements. Reproduced from Ref. [43] with the permission of AIP publishing (License Number: 4457780180478).

Figure 7a,b show the frequency dependences of the real part of the dielectric function, $\varepsilon_{1}$ and the conductivity, $\sigma_{1}$, of the SBR samples with different CB amounts of 0, 15 and 30 wt % under the unstretched condition. To obtain spectra with a wide frequency range, we utilized two impedance analyzers with different measuring ranges and the PS THz-TDS system. In the THz-TDS measurement, the complex dielectric function, $\tilde{\varepsilon }\left(\omega \right)$, is calculated using relation: $\tilde{\varepsilon }\left(\omega \right)=\tilde{N}{\left(\omega \right)}^{2}$. We can obtain $\sigma_{1}$ using the relation presented in Equation (4).

(4) $$\tilde{\varepsilon }\left(\omega \right)=\varepsilon_{1}\left(\omega \right)+i\frac{\sigma_{1\left(\omega \right)}}{\varepsilon_{0}\omega },$$

where *ε*_0_ is the dielectric constant of vacuum. Because a polarization dependence of $\varepsilon_{1}$ and $\sigma_{1}$ under the unstretched condition was not observed in the terahertz frequency range, we only plot one spectrum for each sample. Both figures clearly reveal that the frequency dependences of $\varepsilon_{1}$ and $\sigma_{1}$ for the SBR sample with a CB amount of 30 wt % significantly differ from those for the other samples; in the frequency region below 0.1 MHz, $\varepsilon_{1}$ ($\sigma_{1}$) of the 30 wt % sample is almost three (six) orders of magnitude larger than the $\varepsilon_{1}$ ($\sigma_{1}$) of the samples with lower CB amount. The conductivity of a system that exhibits such a filler-amount threshold behavior is well known and called “percolation conductivity.” In the present material, conductive channels through the entire sample are formed by linking of separate CB aggregates. Since the change in the internal percolating network of conductive channels is quite important, we focus on the samples with 15 and 30 wt %, which correspond to the samples below and above the percolation threshold, respectively. We point out that the measured values for $\varepsilon_{1}$ and $\sigma_{1}$ clearly indicate a continuous connection between the terahertz frequency range and the frequency range measured by the impedance analyzers, which ensures that the dielectric responses of the SBR samples can be appropriately investigated by the terahertz technique.

**Figure 8:**
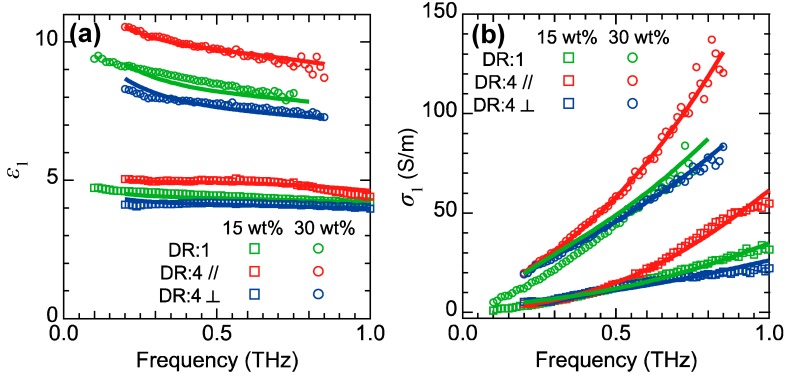
The real part of (**a**) the dielectric function and (**b**) the conductivity for the SBR samples with CB amounts of 15 (squares) and 30 wt % (circles) at DR = 1 (green) and DR = 4 (red and blue). The red and blue symbols correspond to the data parallel and perpendicular to the stretching direction, respectively. The solid curves represent the calculation results. Reproduced from Ref. [43] with the permission of AIP publishing (License Number: 4457780180478).

Figure 8 shows the spectra of $\varepsilon_{1}$ and $\sigma_{1}$ for the SBR samples with CB amounts of 15 and 30 wt % at different DRs. From separate measurements, we confirmed that the $\varepsilon_{1}$ and $\sigma_{1}$ spectra at DR = 4 correspond to the spectra under maximum stretching condition (a full orientation of CB aggregates). Both samples exhibit similar trends. Under the unstretched condition (green data), $\varepsilon_{1}$ monotonically decreases as the frequency increases, while $\sigma_{1}$ monotonically increases. Upon stretching of the samples, an anisotropy appears in the $\varepsilon_{1}$ and $\sigma_{1}$ spectra, where the $\varepsilon_{1}$ and $\sigma_{1}$ spectra parallel (perpendicular) to the stretching direction are larger (smaller) than those under the unstretched condition. This strain-induced change in the $\varepsilon_{1}$ and $\sigma_{1}$ spectra can be attributed to a change in the internal filler condition. 

To derive the change in the internal CB network from the $\varepsilon_{1}$ and $\sigma_{1}$ spectra, we fit these spectra by employing Bruggeman’s effective medium approximation (EMA) [68,69] with the Drude-Smith model [70]. EMA is widely used for interpreting the dielectric functions of mixtures consisting of constituents with different dielectric functions. Here, we assume the same simple mixture model proposed above, that is, each CB aggregate is described by the same rigid and uniaxial ellipsoid. In the EMA model, the dielectric function of the mixture, $\varepsilon_{EMA}$, for perfectly aligned CB aggregates and randomly oriented CB aggregates are described by the following Equations (5) and (6), respectively.

(5) $$f\frac{\varepsilon_{∥\left(⊥\right)}-\varepsilon_{EMA∥\left(⊥\right)}}{g_{∥\left(⊥\right)}\varepsilon_{∥\left(⊥\right)}+\left(1-g_{∥\left(⊥\right)}\right)\varepsilon_{EMA∥\left(⊥\right)}}+3\left(1-f\right)\frac{\varepsilon_{h}-\varepsilon_{EMA∥\left(⊥\right)}}{\varepsilon_{h}+2\varepsilon_{EMA∥\left(⊥\right)}}=0\;$$

(6) $$f\frac{\varepsilon_{∥}-\varepsilon_{EMA}}{g_{∥}\varepsilon_{∥}+\left(1-g_{∥}\right)\varepsilon_{EMA}}+2f\frac{\varepsilon_{⊥}-\varepsilon_{EMA}}{g_{⊥}\varepsilon_{⊥}+\left(1-g_{⊥}\right)\varepsilon_{EMA}}+9\left(1-f\right)\frac{\varepsilon_{h}-\varepsilon_{EMA}}{\varepsilon_{h}+2\varepsilon_{EMA}}=0$$

Here $\varepsilon_{EMA∥\left(⊥\right)}$ and $\varepsilon_{∥\left(⊥\right)}$ are the dielectric functions of the mixture and the CB aggregate parallel (perpendicular) to the orientation direction, respectively. Further, $\varepsilon_{h}$ is the dielectric function of the rubber matrix, $g_{∥\left(⊥\right)}$ is the depolarization factor of the CB aggregates parallel (perpendicular) to the major axis (for a uniaxial ellipsoid, we have $g_{∥}+2g_{⊥}$ = 1) and *f* is the volume fraction of the CB aggregates. Based on a separate measurement on a SBR sample without CB fillers [43], we consider that $\varepsilon_{h}$ is an isotropic and real number (no absorption). Meanwhile, we assume that the dielectric function of the CB ellipsoid is described by the Drude-Smith model [70], which results in Equation (7):

(7) $$\varepsilon_{∥\left(⊥\right)}\left(\omega \right)=\varepsilon_{b}+\frac{i\omega_{p}^{2}\tau_{∥\left(⊥\right)}}{\omega \left(1-i\omega \tau_{∥\left(⊥\right)}\right)}\left(1+\frac{c}{1-i\omega \tau_{∥\left(⊥\right)}}\right),$$

where *ε*_b_ is the background dielectric constant, $\tau_{∥\left(⊥\right)}$ is the scattering time for the direction parallel (perpendicular) to the major axis, *ω*_p_ is the plasma frequency and *c* is a coefficient ranging from −1 to 0, reflecting the delocalization of carriers. For the fitting process, we considered that only the scattering time depends on the direction.

Using Equation (5) and (6), we can fit the $\varepsilon_{1}$ and $\sigma_{1}$ spectra using the relation presented in Equation (4). First, we fitted the $\varepsilon_{1}$ and $\sigma_{1}$ spectra for the stretched condition using Equation (5) and evaluated $\varepsilon_{∥\left(⊥\right)}$, $g_{∥\left(⊥\right)}$ and *f* from the spectra parallel (perpendicular) to the stretching direction, which corresponds to the predominant orientation direction. In this step, we fixed $\varepsilon_{h}$ to 2.43. Then, we reproduced the $\varepsilon_{1}$ and $\sigma_{1}$ spectra under the unstretched condition by substituting the parameters obtained from the stretched condition into Equation (6).

The fitting results are shown with the solid curves in Figure 8. The experimentally obtained polarization-dependent spectra of both samples obtained at DR = 4 are well reproduced by the fitting curves. This means that the dielectric response of the SBR for perfect alignment of the CB aggregates is well described by the simple mixture model based on EMA. However, while the spectra of the SBR with a CB amount of 15 wt % at DR = 1 are well reproduced by the calculated results, those with a CB amount of 30 wt % at DR = 1 are not well reproduced (especially the $\sigma_{1}$ spectrum shows a large deviation at lower frequency region). The good agreement for the 15-wt % sample indicates that the dielectric function of the CB aggregates does not change upon mechanical stretching. On the other hand, the discrepancy between the experiments and calculation in the 30-wt % SBR sample indicates that the dielectric function of the CB aggregates changes upon stretching, which is expected for dense filler networks.

**Figure 9:**
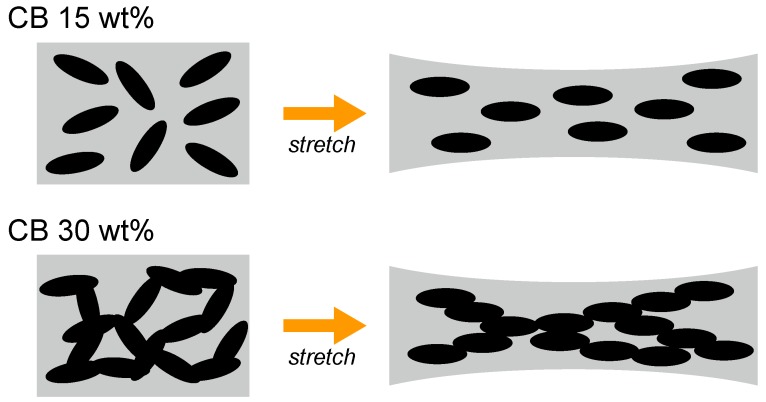
Illustration of the strain-induced change in the internal condition of SBR samples with CB amounts of 15 and 30 wt %.

The above behavior can be intuitively interpreted as illustrated in Figure 9. In case of a SBR sample with a CB amount of 15 wt %, the CB aggregates exhibit a sparse distribution and do not form an internal conductive network. Therefore, each CB aggregate only rotates individually upon stretching. In case of a SBR sample with a CB amount of 30 wt %, the density of the CB aggregates is high enough to form a conductive channels. Here, the percolative network of CB aggregates changes by the strain-induced rearrangement. These results indicate that the anisotropic conductivity measurement based on PS THz-TDS in combination with EMA fitting constitutes an effective tool for investigating the internal conductive CB network under various stretching conditions. 

### 3.4. Discussion

Finally, we briefly discuss the advantages and disadvantages of the PS THz-TDS methods for investigating the internal states of black rubbers compared with the other techniques mentioned in Section 1. First, the PS THz-TDS enables the nondestructive inspection of the internal states of black rubbers in a contactless manners in stark contrast to TEM measurements, which are usually invasive. Second, because the experimental setup of our PS THz-TDS system is compact and portable, one can perform in-situ inspection of rubber products. This is the important advantage with respect to the other techniques for industrial applications. On the other hand, because the wavelength of terahertz light is typically about the millimeter scale, the microscopic structure such as CB aggregates cannot be detected directly different from SAXS and TEM measurements. However, as shown in Section 3.3, with the aid of spectroscopic analysis based on the EMA, the microscopic structure can be evaluated in the PS THz-TDS measurement. As a result, we believed that the PS THz-TDS is an emerging candidate for investigating the internal conditions of the visibly-opaque rubber composites.

## 4. Conclusions

In conclusion, we report on the investigation of the internal status of visibly opaque black rubber composites by a terahertz-based PS spectroscopic technique. It is found that a large birefringence appears in stretched black rubbers with conductive CB fillers. From comprehensive studies including theoretical support from MC simulations that implement a simple mixture model, we revealed that the mechanism of the large birefringence is the strain-induced orientation of anisotropically shaped CB aggregates. With aid of these findings, that is, the strong correlation between birefringence and strain and the validity of the simple mixture model, we succeeded to develop an internal triaxial strain inspection method for black rubbers under arbitrary stretched conditions by means of terahertz polarization spectroscopy. This novel inspection technique has a clear advantage compared to the conventional method based on visible light, because our technique enables inspection of the internal strain, in contrast to the surface-limited information provided by the conventional method. Moreover, we demonstrated that the strain-induced change in the internal conductive CB network can be probed by fitting the dielectric function and conductivity spectra using the EMA theory based on the simple mixture model. Since the internal network plays an important role in the properties of the rubber itself, this nondestructive and noncontact inspection method of the internal network is expected to enable design of more functional rubbers. We emphasize that, although the black rubber composites have a quite complicated structure, the optical responses in the terahertz frequency range can be interpreted by the presented simple mixture model.

In addition, we point out that our technique can be easily employed even in the reflection geometry. Reflection spectroscopy is more convenient for industrial applications when compared to the transmission spectroscopy, because the reflection geometry is easier to handle for operators [38,40,41,44,71,72,73,74]. Very recently, we demonstrated the inspection of the internal filler orientation of black rubbers by PS THz-TDS in reflection mode [75]. However, since the analysis of the reflection signal is more difficult compared to that of the transmission signal, only the angle of the slow optic axis has been obtained but not the degree of birefringence. Because a complete evaluation of the birefringent properties including the degree of birefringence would allow to increase the spectrum of possible applications, further development is required. The demands for polymeric composites (plastics, elastomers) gradually increases due to the improvement of their properties by the effort of many researchers. Thus, we believe that our technique based on the terahertz polarization spectroscopy facilitates a wide variety of researches involving not only fundamental but also applied polymer science.
